# Supplementary figures and images for: Crowdsourcing image analysis for plant phenomics to generate ground truth data for machine learning
Source: PLoS Comput Biol. 2018 Jul 30;14(7):e1006337. doi: 10.1371/journal.pcbi.1006337 (PMC6085066; doi:10.1371/journal.pcbi.1006337)

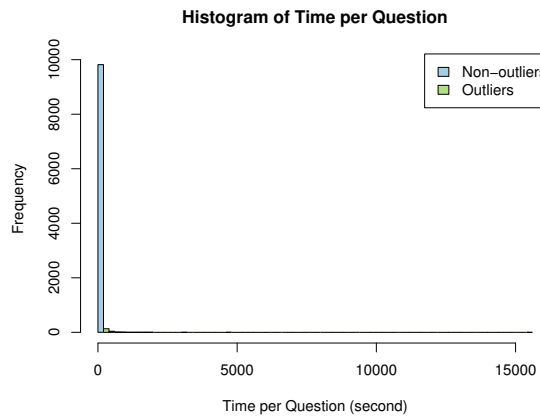

(a)

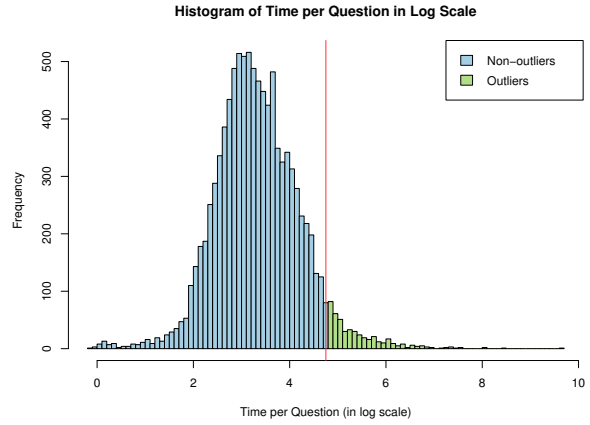

(b)

Histograms of time consumption before and after log transformation.

Supplement: S1 Fig — Outliers in time spent per question, for example 15,484 seconds, were effectively smoothed by log transformation, as shown by the histograms. (PDF) [file pcbi.1006337.s001.pdf]

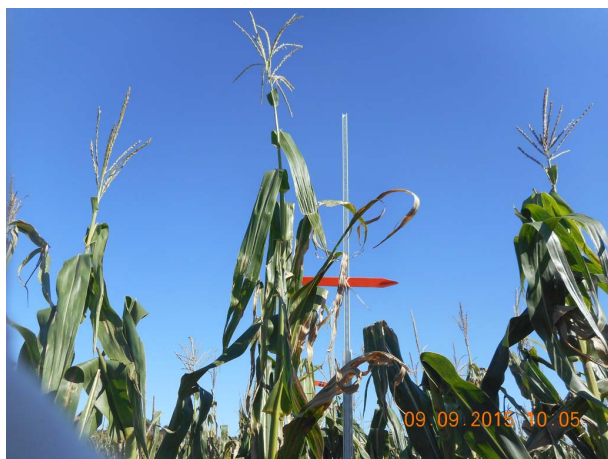

(a) Easy

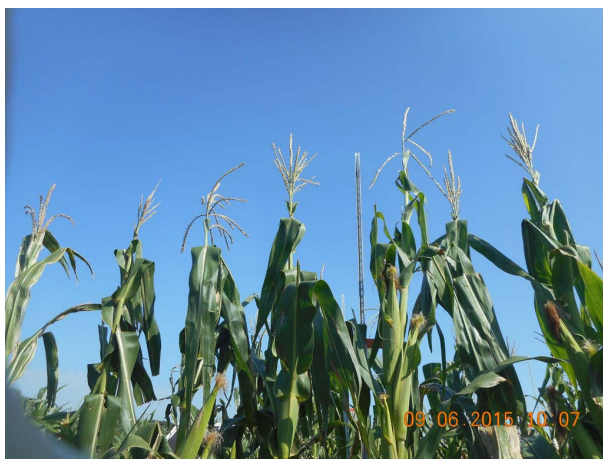

(b) Easy

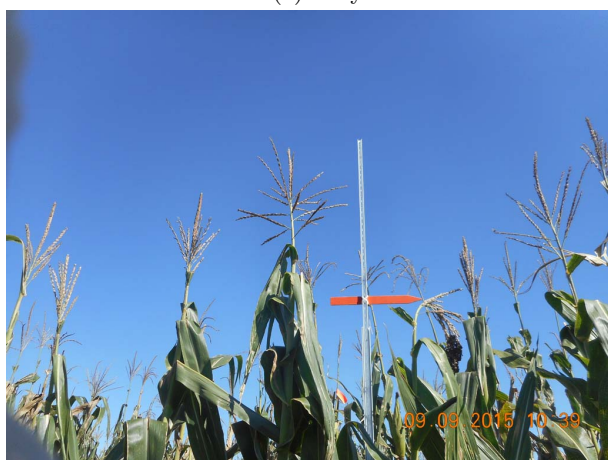

(c) Hard

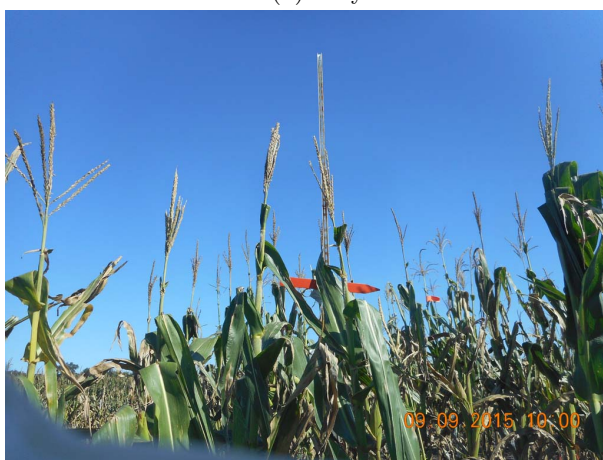

(d) Hard

**Examples of easy and hard images.**

Supplement: S2 Fig — Examples of easy and hard images. In general, hard images have many tassels in the background and overlaps between tassels in the foreground, while easy images have very clear tassels in the foreground. (PDF) [file pcbi.1006337.s002.pdf]
