# Supplementary material for: Crowdsourcing image analysis for plant phenomics to generate ground truth data for machine learning
Source: PLoS Comput Biol. 2018 Jul 30;14(7):e1006337. doi: 10.1371/journal.pcbi.1006337 (PMC6085066; doi:10.1371/journal.pcbi.1006337)
Supplement: S1 Table — Parameter estimates in linear mixed effects regression of accuracy over time. (PDF) [file pcbi.1006337.s003.pdf]

	Estimate ($\hat{\beta}$)	Standard Error	p-value
Master MTurk	-0.00008	0.000038	0.0457
non-Master MTurk	-0.00027	0.000035	< 0.0001
Course Credit	-0.00095	0.000092	< 0.0001

Parameter estimates in linear mixed effects regression of accuracy over time.
