# Supplementary material for: Crowdsourcing image analysis for plant phenomics to generate ground truth data for machine learning
Source: PLoS Comput Biol. 2018 Jul 30;14(7):e1006337. doi: 10.1371/journal.pcbi.1006337 (PMC6085066; doi:10.1371/journal.pcbi.1006337)
Supplement: S1 File — (PDF) [file pcbi.1006337.s004.pdf]

Instructions Given to Participants

Below are the instructions given to the participants in the different groups studied.

Page 1:

We are conducting an academic research survey to help characterize plant features and connect them to the plant genes that influence these features. Participation in this research survey is voluntary and you may skip any questions that you do not wish to answer. Your name and other identifying information will not be collected or connected to your responses. Click the button below to begin the survey.

Page 2:

Technology requirements: To complete this study, you will need to use a laptop or a desktop computer; it will not work properly on a mobile device or tablet. You will also need to use a specific browser: Firefox, Google Chrome or Microsoft's Internet Explorer or Edge; it may not work properly with other browsers.

Overview: In this study, you will be drawing boxes on images of corn plants to identify the locations of corn tassels. This will be useful for researchers developing methods to automate plant analysis.

Example image: Below is an example of an image where the corn tassels have been properly marked. Please examine the image to familiarize yourself with the task.

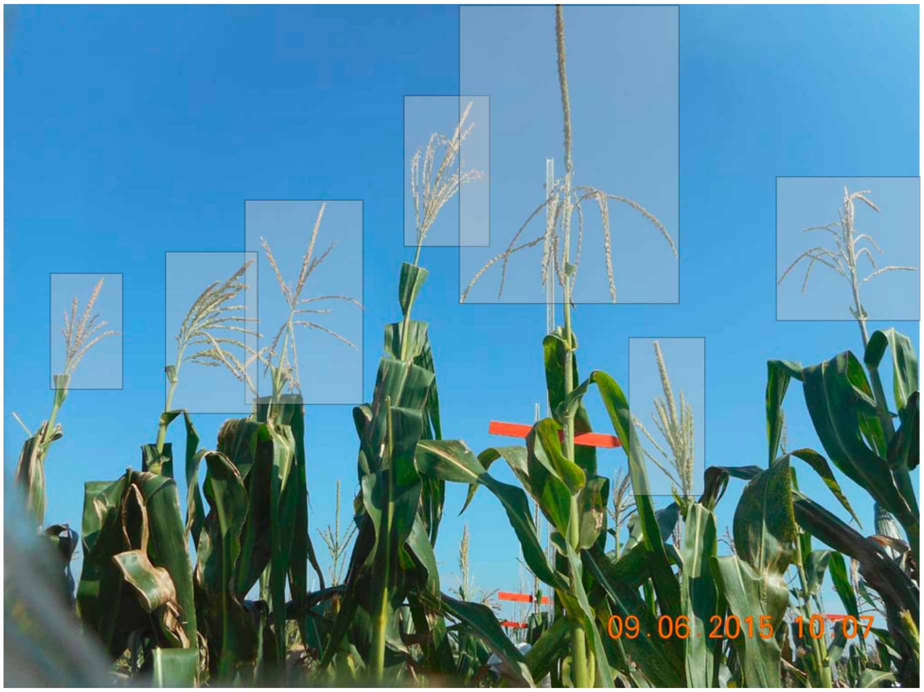

Drawing a box (cropping): Using the computer mouse, click and hold the (left) mouse button to draw a box around part of a photograph containing a corn tassel. Crop as close to the boundaries of each tassel as possible without cutting off any parts of the tassel. When the box you are drawing is properly sized, release the mouse button.

Resizing, moving, or deleting a box: Once you've drawn a box, you can move it by clicking and holding on any non-corner edge of the box. You can also resize the box by clicking and holding on a corner of the box. For both of these operations, release the mouse button when the box is moved or resized to your liking. You can delete a box by clicking once in the middle of the box.

Common issues: The image below has been properly cropped. The numbers (1-3) highlight common issues that you will encounter in this task.

1) Boxes overlap. Its OK if multiple boxes overlap.

2) Extra objects in the box. In this example, there is a metal post behind the tassel. This is OK. Its also OK if there are other things in the box, such as leaves or a bird.

3) Only crop foreground plants (those in the row closest to the camera). Other tassels in the background, such as the one highlighted here, should be ignored.

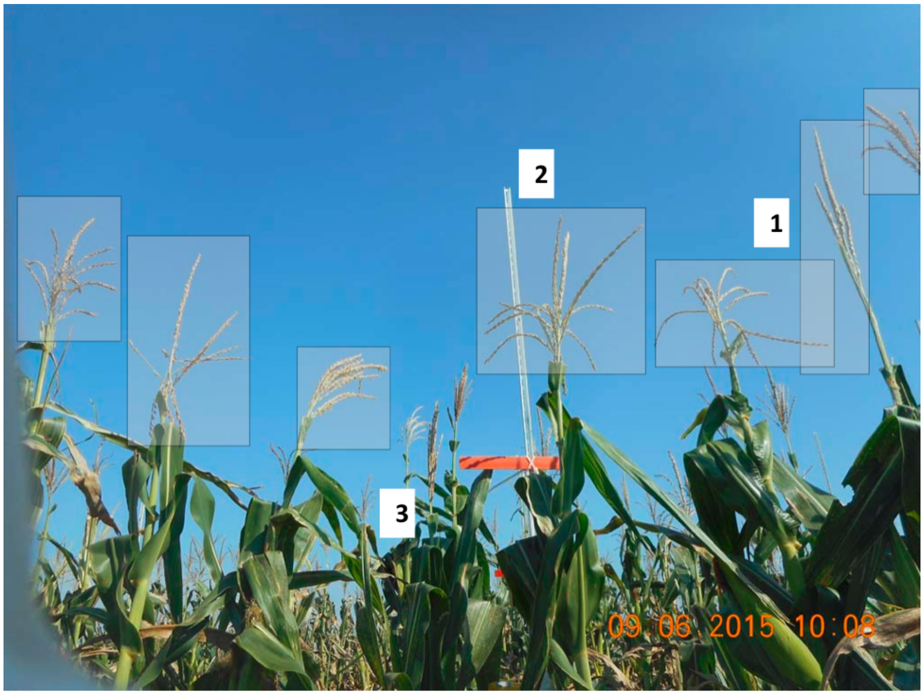

Thank you for your participation.
